# Influenza A (H1N1) Virus Outbreak in the Districts of Chhattisgarh: A Cross-Sectional Study

**DOI:** 10.7759/cureus.55365

**Published:** 2024-03-01

**Authors:** Jaishriram Rathored, Rani Soni, Krishna K Patel, Sandesh Shende, Debashish Samal

**Affiliations:** 1 School of Allied Health Sciences, Central Research Laboratory and Molecular Diagnostics, Datta Meghe Institute of Higher Education and Research, Wardha, IND; 2 Department of Microbiology, Late Baliram Kashyap Memorial Government Medical College, Jagdalpur, IND; 3 Department of Microbiology, Government TCL Postgraduate College, Janjgir, IND

**Keywords:** pandemic, influenza, chhattisgarh, outbreaks, h1n1

## Abstract

Background

The H1N1 flu is a subtype of the influenza A virus, also known as the swine flu. An entirely new strain of the H1N1 virus started sickening people in the 2009-2010 flu season. It was a novel influenza virus combination that can infect humans, pigs, and birds. It was frequently referred to as the “swine flu.” The virus may be able to spread for a little while longer in children and individuals with compromised immune systems.

Objective

The objective is to investigate the outbreaks of H1N1 among young adults in the Bastar District of Chhattisgarh.

Methods

Collection of the blood samples of 342 individuals between December 2015 and November 2017 was done. Thirty-one cases of Influenza A (H1N1) PDM09 virus infection were identified and confirmed. The molecular relationship between viruses is identified by the real-time polymerase chain reaction (RT-PCR) method.

Result

The majority of samples (n=13) were sourced from Raipur Medical College, followed by contributions from Durg District Hospital (n=5), Raigarh Medical College (n=4), Rajnandgaon District Hospital (n=3), Jagdalpur Medical College (n=2), Bilaspur Medical College (n=2), and smaller contributions from Dhamtari District Hospital and Gariyabandh Primary Health Care. Among these, 31 samples tested positive for Influenza A (H1N1) PDM 2009 virus, with a slightly higher prevalence among 19 female patients. Age-wise distribution revealed higher proportions of positive cases in the age groups of 0-10 years, 31-40 years, and 21-30 years. In the molecular analysis, 154 samples showed no target amplification, while 125 samples exhibited amplification of only Influenza A without subtype (H1) amplification. Remarkably, 31 patients who tested positive for Influenza A (H1N1) died from the virus; most of the deaths were in children under five and middle-aged adults.

Conclusion

The detection of Influenza A (H1N1) PDM 2009 virus, especially among females, indicates its persistent circulation. Positive cases were prevalent among younger and middle-aged individuals. Molecular analysis showed subtype variations, with significant fatalities observed in children under five and middle-aged adults, emphasizing the severity of the virus across different age groups. It is advised that in order to keep Indian influenza surveillance up to date and robust, more epidemiological data should be gathered, along with information on risk factors like immunization status, hospitalization, and mortality rates should be estimated, and influenza case subtyping should be improved.

## Introduction

It is likely that a person infected with the virus can transmit it from one day before symptoms manifest to four days after they do. The virus may be able to spread for a little while longer in children and individuals with compromised immune systems. Influenza is a common illness in children with acute lower respiratory infections (ALRI), a common illness that has a substantial effect on healthcare providers globally [1]. Every winter, influenza viruses infect young children, with the youngest age group being most commonly affected [2]. 114,667 cases and 8543 deaths were reported throughout India; this translates to an overall case fatality rate of 7.5% [3]. The predicted mortality from influenza was virtually the same as well. Poisson regression techniques allow for the calculation of the number of fatal crashes linked to influenza A and B, but they also require a substantial amount of viral surveillance data [4]. Influenza, or “flu,” is characterized by acute respiratory illnesses including fever, cough, and malaise, which occur sporadically as seasonal flu, resulting in three to five million severe cases annually around the world [5]. The orthomyxoviridae influenza A and influenza B viruses are spread from person to person. This family of viruses is made up of segmented negative-sense single-strand RNA segments that make up the genome [6].

In India in 2015, the pandemic strain of influenza A H1N1 PDM 2009 caused 27,236 cases and 981 deaths since it first surfaced in April 2009 [7]. Children have been found to be extremely vulnerable to seasonal and pandemic influenza. Early childhood is the main source of community infection and has the highest hospitalization rate. However, how children fit into the influenza pandemic is unknown. Several studies on children have shown that the immunization program decreased the incidence of influenza, underscoring the significance of the communities impacted by global immunization efforts [8]. On April 24, 2009, a virus was found in both Mexico and the United States. The World Health Organization (WHO) informed Member States of this development [9].

Random and inconsistent global influenza A virus pandemic broke out after a novel influenza virus was discovered. A virus strain distinguished from other circulating viruses by the presence of antigens. In general, these viruses originate from animal reservoirs, which are areas where influenza A viruses are common. The investigation of outbreaks involving young adults, as per the current study, lends credence to the possibility that children played a significant role in the transmission of the H1N1 influenza pandemic. and experience higher mortality and morbidity rates due to the absence of previous immunity to the newly acquired virus strain [10,11]. This has important implications for both the 2020-2021 influenza pandemic in the northern part of the world and future influenza vaccine selection.

The current study documents the H1N1 influenza outbreak. In the central Indian state of Chhattisgarh. Samples from acute cases of influenza from different parts of the district during the period from December 2015 to November 2017 were investigated and confirmed in the laboratory of the state VRDL.

## Materials and methods

Study setting

The research study was performed at the Viral Research and Diagnostic Laboratories (VRDL) in Jagdalpur, Chhattisgarh, in the Department of Microbiology at the Late Baliram Kashyap Memorial Government Medical College. The institutional ethics committee approved the study protocol (No/900/GMCJ/ESTT/2015).

Inclusion and exclusion criteria

Among the prerequisites for enrollment are the capacity to live in a Chhattisgarh district, being between the ages of 18 and 65, and exhibiting influenza-like symptoms during the outbreak period. People who have recently left the impacted districts or who have had an influenza vaccination within a year are ineligible. All co-morbidities patients were excluded from the study such as the presence of secondary immunodeficiency states: organ transplantation, diabetes mellitus, malignancy, and treatment with corticosteroids. Patients with HIV-positivity, patients with extrapulmonary TB, and/or patients requiring surgical intervention. Pregnancy and lactation. Patients with a known seizure disorder were also excluded from the study.

Sample size

Since this is an outbreak study, the sample size was not predetermined; instead, all of the samples were collected between December 2015 and November 2017 (while every period is significant to report, the study's main focus is the H1N1 outbreaks in December 2015 and November 2017).

Sample collection

A sample from category C was chosen for real-time polymerase chain reaction (RT-PCR) testing (category C is defined as patients who have breathing difficulties, chest pain, fatigue, hypotension, hemoptysis, and cyanosis. Children exhibiting red flag symptoms of influenza-like illness such as Inability to feed properly, convulsions, dyspnea or respiratory distress, somnolence, high or persistent fever, etc.). This sample was selected as per state IDSP/NCDC guidelines. Between December 2015 and the end of 2017, throat swab samples were obtained using a vacuum tube machine (VTM) and sent to the laboratory from various parts of the state of Chhattisgarh within a day in a cold chain for RT-PCR diagnosis of influenza A H1N1. 

Study population

Four hundred forty-four throat swab samples were collected in total; 342 of those samples were used in the study. The data were sent to the state Integrated Disease Surveillance Project (IDSP) unit and the National Institute of Epidemiology, Chennai (NIE). We received samples from Raipur, Bilaspur, Raigarh, Rajnandgaon, Mahasamund, Dhamtari, Durg, Gariyabandh, Bastar, and other districts of Chhattisgarh. The quality control included NIV training of VRDL staff and sending 10% of samples to NIV, Pune for proficiency testing.

Laboratory analysis

RNA was extracted using the Thermo-Fisher Scientific Invitrogen viral RNA/DNA mini kit, and the SuperScript® III Platinum® One-Step Quantitative RT-PCR System (Invitrogen Life Technologies) was used for the RT-PCR super mix in compliance with the manufacturer's instructions.

Amplification of the desired gene by RT-PCR

The Influenza A (H1N1) Primer and Probe Set contains the 12 primers and probes listed below. These primers and probes match the WHO/CDC Protocol titled “CDC protocol of real-time (RTPCR) for swine influenza A(H1N1).”

Inf A Forward GAC CRA TCC TGT CAC CTC TGA C; Inf A Reverse AGG GCA TTY TGG ACA AAK CGT CTA; InfA Probe1 TGC AGT CCT CGC TCA CTG GGC ACG; SW InfA Forward GCA CGG TCA GCA CTT ATY CTR AG; SW InfA Reverse GTG RGC TGG GTT TTC ATT TGG TC; SW InfA Probe2 CYA CTG CAA GCC CA”T” ACA CAC AAG CAG GCA; SW H1 Forward GTG CTA TAA ACA CCA GCC TYC CA; SW H1 Reverse CGG GAT ATT CCT TAA TCC TGT RGC; SW H1 Probe2 CA GAA TAT ACA “T”CC RGT CAC AAT TGG ARA A; RnaseP Forward AGA TTT GGA CCT GCG AGC G; RnaseP Reverse GAG CGG CTG TCT CCA CAA GT; RnaseP Probe 1 TTC TGA CCT GAA GGC TCT GCG CG. TaqMan® probes are labeled at the 5’-end with the reporter molecule 6-carboxyfluorescein (FAMTM) and with a quencher moiety at the 3’-end. TaqMan® probes are labeled at the 5’-end with the reporter molecule 6-carboxyfluorescein (FAMTM) and quenched internally at a modified “T” residue with a quencher moiety, with a modified 3’-end to prevent probe extension by Taq polymerase.

The extracted RNA is amplified in ABI 7500 fast (Applied Biosystems™ 7500 Real-Time PCR Systems) using Kit and reagent supplied by Invitrogen and as per manufacturer instructions. One cycle of reverse transcription for DNA polymerase activation and reverse transcriptase inactivation made up the thermal profile. It lasted 10 minutes at 45 °C and 10 minutes at 95 °C, respectively. 45 cycles of denaturation (at 95 °C for 15 seconds), annealing (at 54 °C for one minute), and extension (at 72 °C for 15 seconds) were used to amplify the cDNA. The annealing step of the reaction was when the fluorescent signal peaked, and sequence detector software (7500 System Software v.1.3.1, Applied) was used to analyze the data. The average of the duplicates for each standard and sample is displayed in the data.

Statistical analysis

The results were analyzed for descriptive study considering the frequency and percentage of outcome evaluation generated for the H1N1 incidence using the SPSS software version 2017 (IBM Corp., Armonk, NY). The incidence of H1N1 for gender amongst males and females and age group distribution was calculated using Chi-square analysis and found significant for gender as p-value < =0.05.

## Results

**Figure 1:**
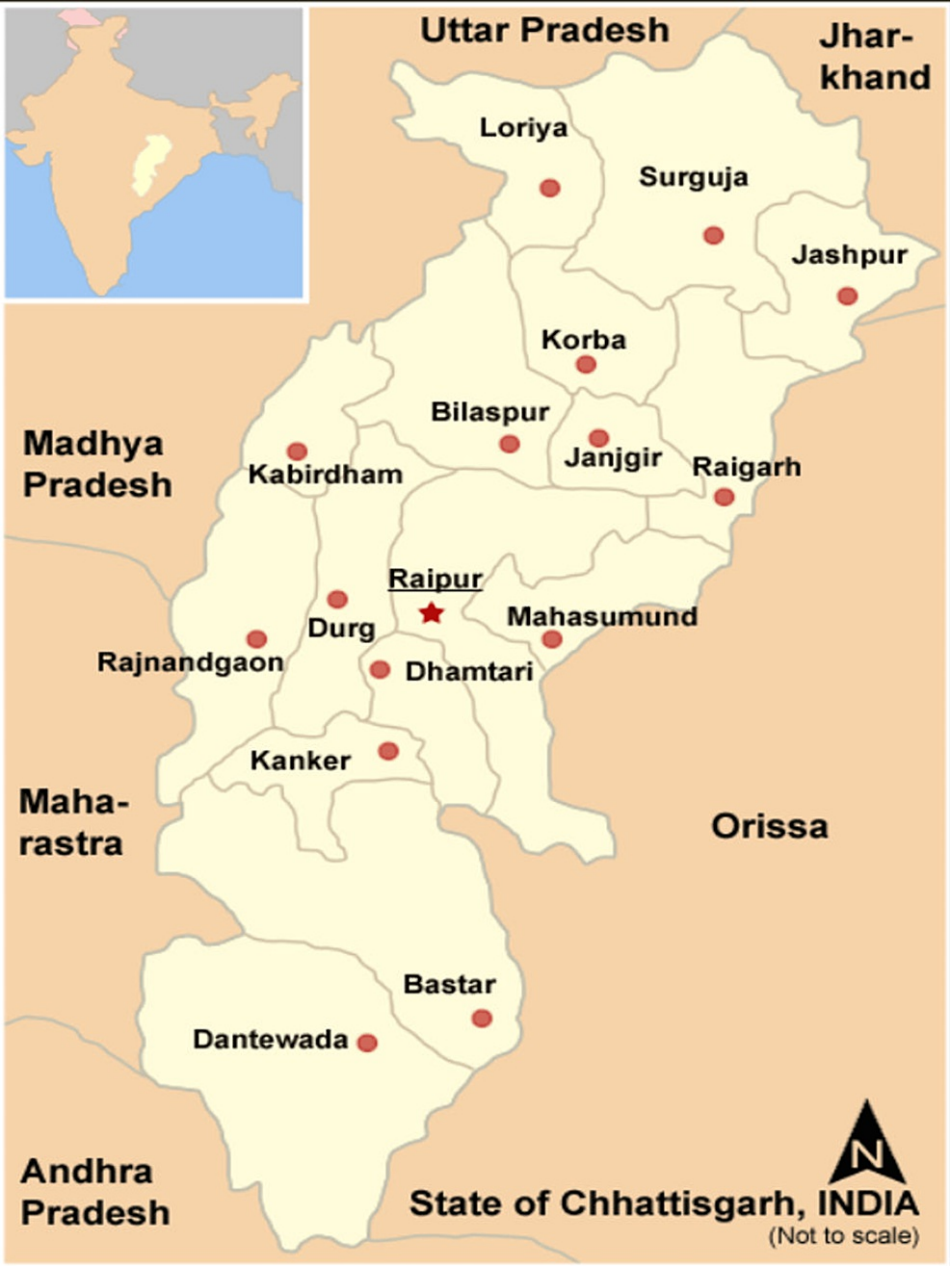
A map of Chhattisgarh showed the districts affected by the outbreak The "Map Chhattisgarh state and districts.png" file was downloaded from open-source website. The number 19 was used to make the reference.

Raipur Medical College (n=13), Durg District Hospital (n=5), Raigarh Medical College (n=4), Rajnandgaon District Hospital (n=3), Jagdalpur Medical College (n=2) Bilaspur Medical College (n=2), Dhamtari District Hospital, and Gariyabandh Primary Health Care each sent a sample of critically ill patients. The outbreak districts in Chhattisgarh are shown in Figure 1.

Between December 2015 and November 2017, 444 throat swab samples in VTM were received for laboratory diagnosis of influenza. After it was established that 342 of these samples were category C patients, they were included in the study's participant pool. Thirty-one (9%) samples were identified as influenza A H1N1 of these 12 samples (38.2%) were from male patients and 19 (61.2%) were from female patients and found statistically significant (p<0.039) (Table 1).

**Table 1:** Frequency distribution of gender for H1N1 incidence H1N1 is a subtype of influenza A virus. The H stands for hemagglutinin, and the N stands for neuraminidase.

Gender	Positive	Negative	Chi-square (p value)
Male	12	198	4.252 (0.039)
Female	19	144	

Only influenza A was amplified (H1subtype not amplified) in 125 (28.1%) samples, while no target was amplified in 154 (34.6%) samples. Eleven of the 31 patients (35.4%) who tested positive for H1N1 and influenza A passed away. Of these, one was 67 years old, five were middle-aged adults, and five were children (45.4%). The age distribution of influenza A H1N1 patients is shown in Table 2, and no statistically significant results were observed (p<0.957).

**Table 2:** Age wise distribution of influenza A H1N1 positive patients H1N1-H1N1 is a subtype of influenza A virus. The H stands for hemagglutinin, and the N stands for neuraminidase, PDM-2009-Pandemic Disease Mexico 2009

Age group	Total number of samples	Influenza A(H1N1) PDM 2009 positive	Chi-square (P value)
0-10	67	7 (22.5 %)	2.047 (0.957)
11-20	33	3 (9.6 %)	
21-30	78	6 (19.3 %)	
31-40	66	7 (22.5 %)	
41-50	52	4 (12.9 %)	
51-60	20	3 (9.6 %)	
61-70	22	1(3.2 %)	
71-80	4	0	

When compared to other districts in Chhattisgarh, Raipur has the highest attack rate and Mahasamund the lowest (Table 3).

**Table 3:** Attack rate (outbreak) of influenza A H1N1 positive patients district wise H1N1 is a subtype of influenza A virus. The "H" stands for hemagglutinin, and the "N" stands for neuraminidases.

Districts of Chhattisgarh	Total no. of samples (n)	Positive cases (n)	Percentage (%)	Total population (n)	Attack rate (%)
Raipur	161	13	41.9	1010087	0.00128
Bilaspur	17	2	6.45	2662077	0.00007
Raigarh	31	4	12.90	150000	0.00266
Mahasamund	17	0	0	1032754	0
Dhamtari	11	1	3.22	799199	0.00012
Durg	28	5	16.12	268806	0.00186
Gariayabandh	8	1	3.22	10517	0.00950
Bastar	23	2	6.45	1411644	0.00014
Rajnandgaon	12	3	9.67	163000	0.00184
others	34	0	0	Not available	0
Total	342	31	100	7508084	0.00041

The rate of influenza case fatalities (outbreak) at the district level An H1N1 positive patient is shown in Table 4, where the highest rate is found in Raipur and the lowest rate is found in Mahasamund. For other districts, comparable outcomes were found.

**Table 4:** Case fatality rate (outbreak) of influenza A H1N1 Positive patients district wise H1N1 is a subtype of influenza A virus. The "H" stands for hemagglutinin, and the "N" stands for neuraminidases.

Districts of Chhattisgarh	Positive cases (n)	Case fatality rate (%)
Raipur	13	61.53
Bilaspur	2	50
Raigarh	4	0
Mahasamund	0	0
Dhamteri	1	0
Durg	5	40
Garia yabandh	1	0
Bastar	2	0
Rajnandgaon	3	0
others	0	0
Overall case fatality rate	31	35.48

## Discussion

VRDL Jagdalpur performs diagnostic tests for virus infection in the state of Chhattisgarh in coordination with IDSP and the National Vector-Born Disease Control Program (NVBDCP). If the pandemic virus infects swine, it is critical to assess a specific new diagnostic instrument that allows for the timely detection of the pandemic (H1N1) in pigs. The development of precise diagnostic tests is crucial to the effectiveness of response strategies to swine outbreaks in reducing the risk of infection in humans [12-15]. The 2009 swine flu pandemic was the third known pandemic involving the H1N1 virus, the other two being the 1977 Russian flu and the 1918-1920 Spanish flu pandemic. The 2009 pandemic was caused by the H1N1/swine flu/influenza virus and was declared by the World Health Organization (WHO) from June 2009 to August 2010 [5]. The outbreak of pandemic influenza H1N1 PDM 09 created panic across the globe. India also experienced the brunt. Sporadic cases due to influenza H1N1 PDM continued in several parts of India. The present study reports an outbreak of H1N1 PDM in central India from 2015 to 2017. In Chhattisgarh, the prevalence of human influenza virus infection is similar to that of other Indian states. During the wet season, sample collections were higher, as stated in India's national health profile. Of these samples, 7.9% tested positive for influenza viruses belonging to the H1N1, H1 subtype, and Influenza-A subtypes. Most of the positive samples were influenza A H1N1 PDM 2009 strains. Eleven of the 31 (35.4%) patients died during treatment, and most of them were children and middle-aged adults. Only one of the victims was over 65 years old. Five of the 11 fatalities were children below five years old, and five were between 30 and 65 years old, and this probably reflects a lack of prior exposure to influenza A (H1N1). To treat the human influenza virus, the infected individual must be isolated, and treated with antiviral medication, and any further complications must be treated. Antiviral neuraminidase inhibitors (NAIs) such as Oseltamivir, Zanamivir, Peramivir, and Laninamivir, as well as amantanes like amantadine and rimantadine, are the mainstays of therapy; however, when treatment is initiated within 12 hours of the onset of symptoms, oseltamivir, also known as Tamiflu, is very effective in reducing the incidence of otitis media in children aged one to three years. It is the most commonly prescribed oral medication for swine flu and seasonal influenza [16,17]. According to studies reported by Akano et al., breathlessness, coughing, fever, and sore throats were the typical swine flu symptoms. Common findings included decreased levels of procalcitonin in blood, low oxygen saturation (hypoxia), elevated respiratory rate (tachypnea), and a WBC count within normal limits. Therefore, we should suspect swine flu in patients with pneumonia who present with the aforementioned symptoms during the winter, especially in nations like India, and we must rationalize their care as soon as possible. Gender had no discernible impact on the distribution of human influenza cases in the current study, However, Raipur, the state capital, has seen higher incidence rates than other surrounding districts such as Durg and Raigarh. All of the samples that were received showed that influenza A was more common followed by influenza B, C, and D. The worst results were seen in patients who required invasive ventilation. Therefore, the chances of survival for swine flu patients with severe pneumonia, hypoxemia, and lung injuries requiring invasive support decreased significantly. However, these outcomes could have been avoided with early use of healthcare resources and appropriate preventive measures [18] Influenza is still a major cause of respiratory infections, particularly in young people. Although there is a seasonal variation in the number of influenza cases throughout the year, the dry season sees more cases than the wet season. We recommend standard and droplet precautions for all residents in long-term care facilities who have influenza, regardless of whether the illness is suspected or confirmed. Furthermore, we also, recommend that all adults, healthy or not, who are six months of age or older, get an annual influenza vaccination.

One of the limitations of this study is the non-availability of dry ice, delay in transit, and throat swabs probably degraded RNAs in as many as 21 samples as evidenced the by failure to amplify RNAse P housekeeping gene. Another limitation is most often samples were received in two to four days, but a few shipments were delayed as long as 11 days. Of the 444 samples from various parts of the Chhattisgarh states [19], 342 belong to category C influenza patients and in this 125 (28.1%) only influenza A was amplified (H1N1 negative) probably representing other strains of seasonal flu. In 2020, many tropical Asian nations are experiencing difficulties undertaking influenza surveillance, most likely as a result of COVID-19-related disruptions. Similar to what has been noticed across the Southern Hemisphere [12-14] flu continues to spread, and some regions have seen an extended return of the viral activity in their communities. Conversely, a lot of empathies rely on publicly accessible research. The sample sizes of each study vary, which reflects the precise estimation of the population attribute; however, in various age groups, they have been over- or under-estimated. The incidence rate of contact with children is crucial for the virus's transmission in children, according to a 2009 H1N1 study [20]. As a result, childhood vaccination has been widely adopted and acknowledged as a successful means of lowering the death rate and stopping the spread of the 2009 H1N1 [21]. Global surveillance and notification systems data indicate that individuals with underlying medical or clinical conditions, those under the age of two, and those who are morbidly obese are at an increased risk of contracting H1N1. Moreover, low serum IgG2 levels were linked, mostly in pregnant women, to the severity of the 2009 H1N1 infection [22].

The observed variation in the level of activity related to influenza among tropical Asian nations is significant and could be attributed to various factors such as the degree of restrictions on travel, artefacts in surveillance, and compliance with COVID-19 recommendations.

## Conclusions

In the present study, the state capital had higher incidences than nearby districts like Raigarh and Durg and gender did not significantly affect the distribution of human influenza cases; however, influenza A was more common than H1N1 and H1 subtype in all samples that were received. In conclusion when other major outbreaks occur, such as the COVID-19 pandemic, disruptions can be avoided by keeping an eye out for integrated pan-respiratory diseases. As a result, it is suggested that Indian influenza surveillance be maintained current and strengthened by gathering more epidemiological information, risk factors like immunization status, estimating influenza-related outcomes like hospitalizations and mortality, and improving influenza case subtyping. Further qualitative research should be carried out to determine the obstacles to completing data collection. This will enhance vaccination programs and enable early identification and response to influenza epidemics or pandemics.
